# Mitochondrial stress and mitokines in aging

**DOI:** 10.1111/acel.13770

**Published:** 2023-01-15

**Authors:** Johannes Burtscher, Afsaneh Soltany, Nishant P. Visavadiya, Martin Burtscher, Grégoire P. Millet, Kayvan Khoramipour, Andy V. Khamoui

**Affiliations:** ^1^ Institute of Sport Sciences University of Lausanne Lausanne Switzerland; ^2^ Department of Biomedical Sciences University of Lausanne Lausanne Switzerland; ^3^ Department of Biology, Faculty of Science University of Shiraz Shiraz Iran; ^4^ Department of Exercise Science and Health Promotion Florida Atlantic University Boca Raton Florida USA; ^5^ Department of Sport Science University of Innsbruck Innsbruck Austria; ^6^ Department of Physiology and Pharmacology, Neuroscience Research Center, Institute of Neuropharmacology, and Afzalipour School of Medicine Kerman University of Medical Sciences Kerman Iran

**Keywords:** FGF21, GDF15, humanin, mitochondria‐derived peptides, mitochondrial stress response, mitohormesis, mitokines

## Abstract

Mitokines are signaling molecules that enable communication of local mitochondrial stress to other mitochondria in distant cells and tissues. Among those molecules are FGF21, GDF15 (both expressed in the nucleus) and several mitochondrial‐derived peptides, including humanin. Their responsiveness to mitochondrial stress induces mitokine‐signaling in response for example to exercise, following mitochondrial challenges in skeletal muscle. Such signaling is emerging as an important mediator of exercise‐derived and dietary strategy‐related molecular and systemic health benefits, including healthy aging. A compensatory increase in mitokine synthesis and secretion could preserve mitochondrial function and overall cellular vitality. Conversely, resistance against mitokine actions may also develop. Alterations of mitokine‐levels, and therefore of mitokine‐related inter‐tissue cross talk, are associated with general aging processes and could influence the development of age‐related chronic metabolic, cardiovascular and neurological diseases; whether these changes contribute to aging or represent “rescue factors” remains to be conclusively shown. The aim of the present review is to summarize the expanding knowledge on mitokines, the potential to modulate them by lifestyle and their involvement in aging and age‐related diseases. We highlight the importance of well‐balanced mitokine‐levels, the preventive and therapeutic properties of maintaining mitokine homeostasis and sensitivity of mitokine signaling but also the risks arising from the dysregulation of mitokines. While reduced mitokine levels may impair inter‐organ crosstalk, also excessive mitokine concentrations can have deleterious consequences and are associated with conditions such as cancer and heart failure. Preservation of healthy mitokine signaling levels can be achieved by regular exercise and is associated with an increased lifespan.

## Graphical abstract

Mitokines signal mitochondrial stress to distant cells and tissues. Long‐lasting increases or decreases in mitokine levels are associated with diseases and age‐related functional decline. Controlled fluctuations of mitokines, such as in exercise, may improve mitokine sensitivity and promote healthy aging.

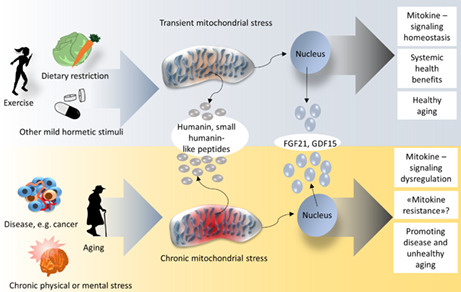

## 1 INTRODUCTION

Mitochondria form dynamic networks in cells and exchange molecules and information among each other, with the cytoplasm and with other organelles (Vincent et al., 2017). They are increasingly recognized to be able to communicate across cellular boundaries and even across tissues, although the precise roles of mitochondria in such inter‐tissular communication and the consequences on health and disease are not yet well understood. A prominent example for inter‐tissue crosstalk at least partially mediated by mitochondria is the communication of exercise signals from skeletal muscles to various organs, including the brain (Benarroch, 2022; Burtscher, Millet, et al., 2021; Thyfault & Bergouignan, 2020).

The present review aims to summarize recent research on mitokines and their actions, their regulation by exercise and other lifestyle interventions and consequences on aging. We discuss current knowledge gaps on how mitokines act and the interplay with other mitochondrial communication channels. We highlight how inter‐mitochondrial information exchange can lead from tissue‐restricted mitochondrial stress to either the initiation of beneficial, healthy aging‐promoting cellular and systemic adaptations or pathological processes through the modification of mitochondrial populations in remote tissues and organs. Special emphasis is put on the risk of the dysregulation of mitokine‐signaling and the associated facilitation of age‐related diseases, and the potential of exercise to maintain balanced mitochondrial stress‐mediated communication.

## 2 MULTI‐ORGAN RESPONSES TO THE MITOCHONDRIAL STRESS RESPONSE

Mitochondrial insults, including oxidative stress, unfolded proteins and impairment of the electron transport system, all eventually impairing mitochondrial protein import, trigger a mitochondrial stress response, which coordinates an array of adaptive responses (Mottis et al., 2019; Shpilka & Haynes, 2018). This stress response and related adaptations are major determinants of the aging process, as recently reviewed (Rose et al., 2017). An important part of the mitochondrial stress response is the mitochondrial unfolded protein response, which signals proteotoxic stress and induces protective adaptations, such as metabolic reprogramming and epigenetic remodeling (Boos et al., 2020; Mottis et al., 2019; Zhou et al., 2022). It is considered a retrograde response, since the signaling originates from mitochondria and represents a mito‐nuclear feedback mechanism (Rose et al., 2017). Among the targets of the mitochondrial stress response are the regulation of mitochondrial import, mitochondrial and cytoplasmic proteostasis, recovery of oxidative phosphorylation (OXPHOS), and the clearance of defective mitochondria, termed mitophagy (Boos et al., 2020; Mottis et al., 2019; Shpilka & Haynes, 2018). Beside the unfolded protein response, other mechanisms also contribute to cellular stress adaptations and include translation initiation factor eIF2α‐mediated reduction of global protein synthesis and the heat shock response (Shpilka & Haynes, 2018).

**FIGURE 1:**
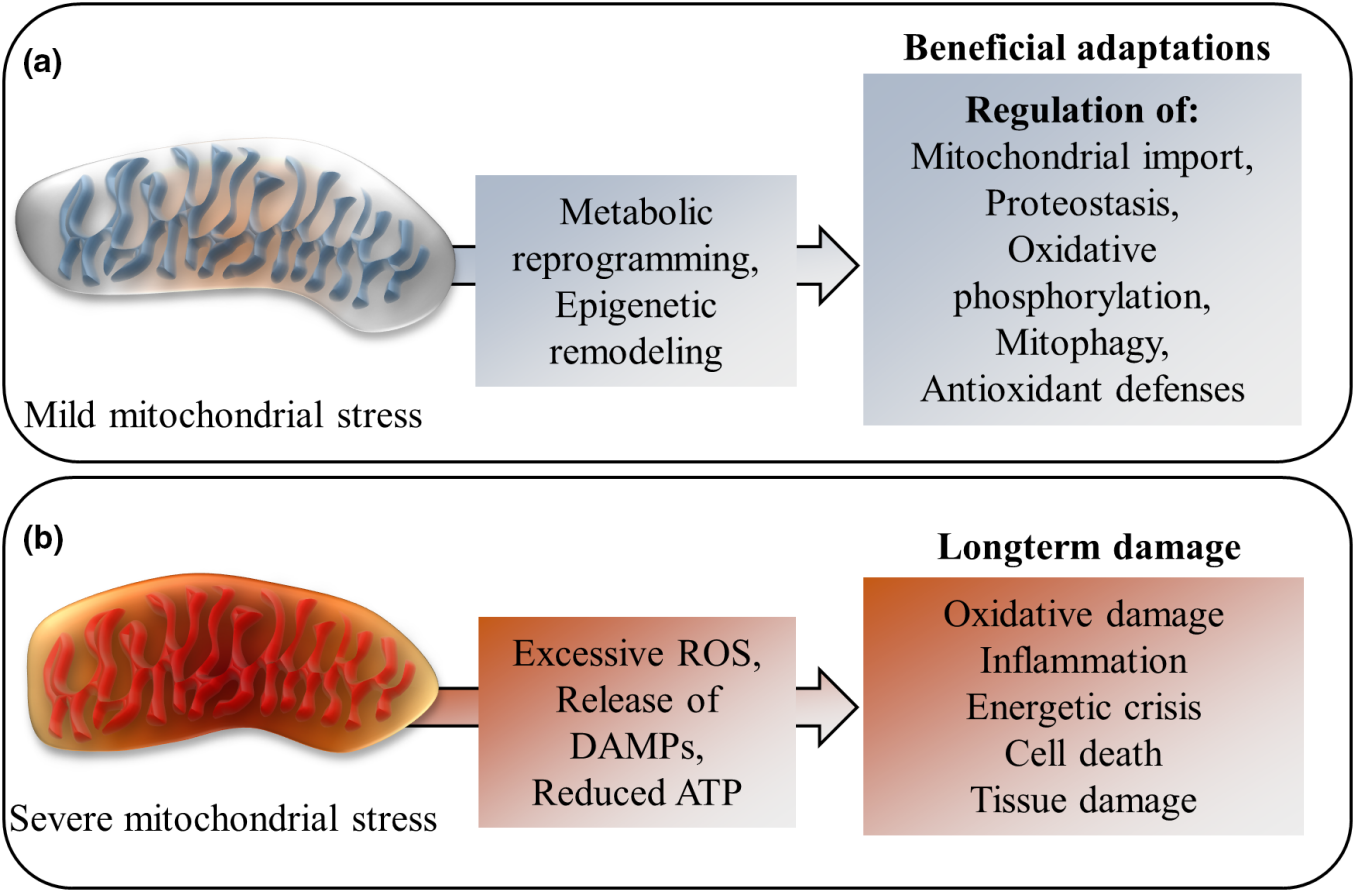
The biphasic response to mitochondrial stress. (a) Mild mitochondrial stress in a healthy organ and organisms with sufficient adaptive capacities will lead to beneficial adaptations both on the cellular and systemic level. (b) Conversely, severe mitochondrial stress or a compromised adaptation capacity may result in the production and release of excessive ROS, mitochondrial damage associated molecular patterns (DAMPs) and reduced levels of energy, leading to permanent cellular and tissue damage

The mitochondrial stress response is a hormesis phenomenon (“mitohormesis” (Yun & Finkel, 2014)) with a biphasic response: while mild mitochondrial stress stimulates cytoprotective adaptations, including by inducing responses of the nucleus and various cytosolic proteostasis preserving pathways (D'Amico et al., 2017), severe mitochondrial stress is harmful (Figure 1). These dose‐dependent mitohormesis effects are determined for example by the concentration of mitochondrial reactive oxygen species (ROS) or the severity of reduced oxygen availability (hypoxia), which either induce beneficial adaptations (if the stress is mild) or damage (in cases of severe stress; Burtscher, Mallet, et al., 2022; Ristow & Schmeisser, 2014).

The mitochondrial stress response between multiple organ systems can be induced in experimental model systems, for example, by partial inhibition of components of the electron transport system. This approach has been demonstrated to cause mitochondrial stress responses in distal tissues (e.g., in the intestine) following mitochondrial perturbation in specific tissues (e.g., in neurons) in *C. elegans* that results in systemic effects, like increased longevity (Durieux et al., 2011). Based on these observations, a cell non‐autonomous signal upon sensing of the mitochondrial stress was assumed to travel to other tissues to induce physiological responses. This then un‐characterized signal was termed a “mitokine” (Durieux et al., 2011).

The best investigated naturally occurring mitochondrial stress that leads to systemic benefits, is mitochondrial strain in skeletal muscle by exercise, as recently reviewed (Burtscher, Millet, et al., 2021). Mechanical stress in skeletal muscle in combination with local changes in temperature, oxygen consumption and metabolism exert pronounced systemic effects on numerous organ systems, including the cardiovascular, respiratory, immune and nervous systems. Global physical and biochemical changes (e.g., temperature, pH), blood flow alterations, and signaling via numerous circulatory factors contribute to mediate these effects (Burtscher, Burtscher, & Millet, 2021; Burtscher, Millet, et al., 2021). Among these mediators are mitokines that are released upon mitochondrial stress and likely act according to mitohormetic principles.

One important stressor and cellular cue for mitochondrial adaptations in physical exercise in muscle is hypoxia. The dependence of mitochondrial OXPHOS on oxygen renders mitochondria particularly vulnerable to hypoxia. Therefore, cells need to adapt to hypoxia quickly and they do this via several mechanisms that notably include the transcription factor family hypoxia inducible factors that are involved in hypoxia‐linked cellular responses. These include the induction of cell‐protective pathways and metabolic programming, leading to more efficient oxygen supply and utilization (Burtscher, Mallet, et al., 2022). Efficient adaptations to hypoxia confer health benefits and can increase longevity, as recently reviewed (Burtscher, Mallet, et al., 2021).

The aging process by itself is further associated with progressively reduced mitochondrial functions (Panel et al., 2018) and many age‐related diseases, including cancers, metabolic diseases, and neurodegenerative disorders, are characterized by mitochondrial deficits (Murphy & Hartley, 2018; Wallace, 2012). How these impairments mechanistically influence aging and age‐related disease progression remains to be fully elucidated. But inter‐mitochondrial signaling—via mitokines and other signaling molecules—is an important factor, as will be outlined below.

## 3 INTER‐MITOCHONDRIAL COMMUNICATION MODES

**FIGURE 2:**
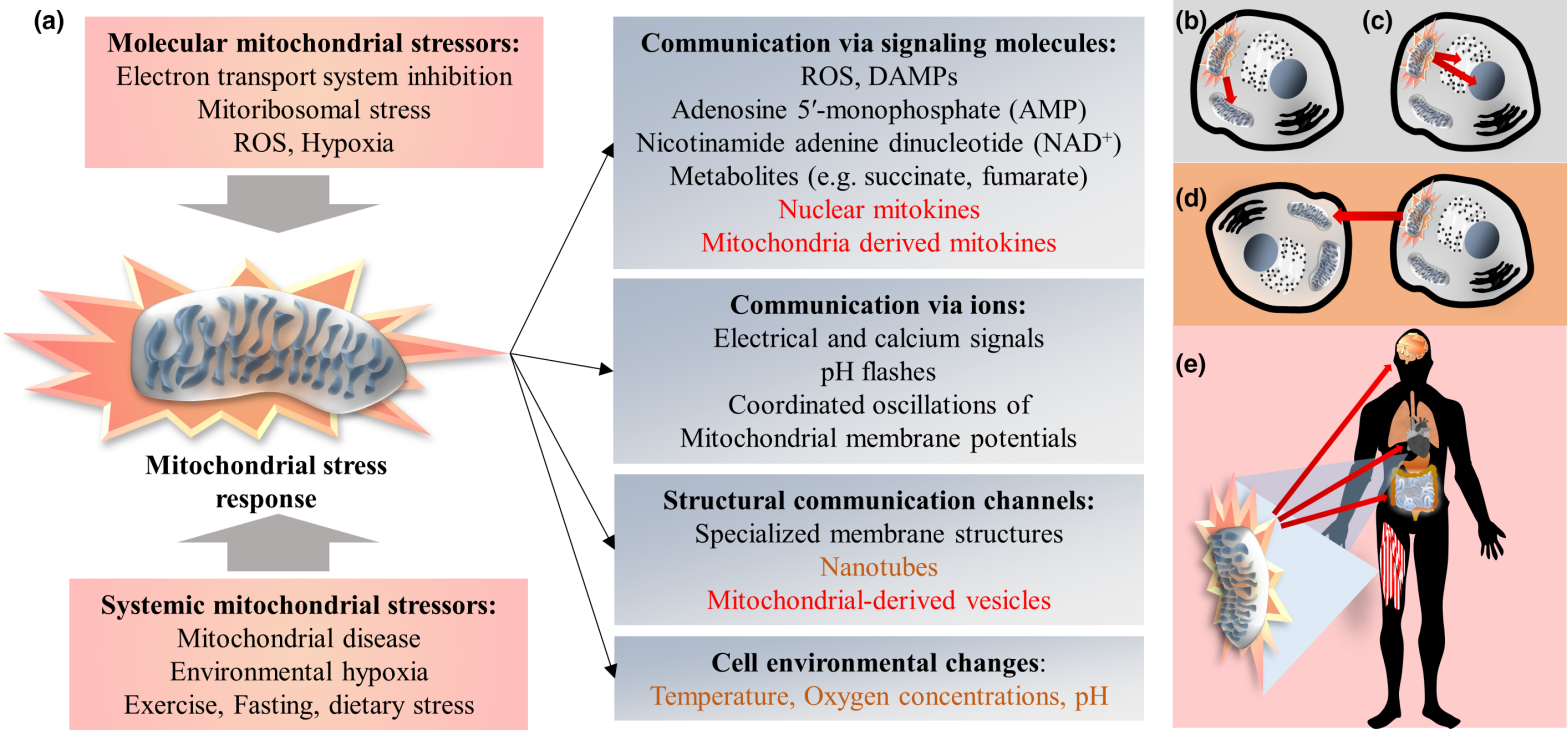
Mitochondrial communication of mitochondrial stress. (a) Various molecular stressors in response to systemic or cellular/cell environmental cues can trigger the mitochondrial stress response, which is transmitted via various communication channels. The stress responses can be communicated to other mitochondria (b), other organelles within the cell (c), to other cells (d) or to distant tissues (e). While moderate mitochondrial stress can lead to beneficial adaptations, severe mitochondrial stress or a compromised adaptation capacity may result in the production and release of excessive ROSs, mitochondrial DAMPs, inflammation, reduced levels of energy and ultimately to permanent cellular and tissue damage

Mitochondrial stress may be propagated through several different modes of communication (Figure 2).

Mitochondrial communication within cells has been described as a “mitocellular communication network” based on numerous “metabolic languages” (Mottis et al., 2019), with mitochondria sending and receiving a variety of signals to and from other organelles and the cytosol. Particularly mito‐nuclear information exchange has been highlighted as a determinant of mitochondrial function, the cellular capacity to respond to various challenges and ultimately of aging and longevity (Rose et al., 2017). Beside the crosstalk via physical contact or intracellular signaling molecules (for reviews see Mottis et al. (2019) and Rose et al. (2017)), mitochondria also make use of extracellular messaging leading to non‐cell‐autonomous effects. The ability of mitochondria to signal mitochondrial stress to distant tissues and induce resilience‐enhancing adaptations also there, contribute to systemic benefits following local, mild mitochondrial stress (Rose et al., 2017). The joint system of mitochondria and other associated cellular components that sense, integrate and signal cellular status has recently been termed the “mitochondrial information processing system” (Picard & Shirihai, 2022).

Many potential mechanisms have been proposed by which mitochondria transfer signals across cellular boundaries. These may be indirect by mitochondria‐mediated modulation of systemic or cell‐environmental factors, such as temperature, local oxygen concentrations or pH (Burtscher, Millet, et al., 2021), which in addition are involved in the sensing of metabolic information by mitochondria. While some other communication channels rely on diffusion or traditional facilitated transport of signaling molecules, also various structural cellular components contribute to inter‐mitochondrial communication, such as nanotubes (Vincent et al., 2017), mitochondrial‐derived vesicles (Sugiura et al., 2014), as well as specialized mitochondrial membrane structures that are involved in the inter‐organelle coordination of cristae (Picard et al., 2015).

Circulating factors that can improve systemic functions and resilience are intriguing communication channels and under intense investigation, especially since Conboy and colleagues demonstrated in heterochronic parabiosis studies that young blood could restore the regenerative capacity of aged satellite cells (Conboy et al., 2005). Some of these factors likely signal mitochondrial stress and induce beneficial adaptations in distant tissues (Rose et al., 2017) and they may be soluble or transported in extracellular vesicles.

Extracellular vesicles are heterogeneously sized lipid‐bilayer delimited vesicles secreted by most cells. They are important vehicles for inter‐cellular communication by delivering molecules, such as proteins, lipids, carbohydrates and nucleic acids (Saludas et al., 2021), and include signaling molecules that have the potential to modulate mitochondrial functions of target cells. One example of the exchange of a protein that leads to improved bioenergetics is extracellular vesicle‐dependent transport of the nicotinamide adenine dinucleotide (NAD)‐dependent deacetylase sirtuin 2 from oligodendrocytes to neuronal axons, leading to enhanced deacetylation of mitochondrial proteins and resultant improved ATP‐production (Chamberlain et al., 2021).

While many molecules can be shuttled in smaller extracellular vesicles (usually 40–100 nm in diameter), larger extracellular vesicles of diameters >200 nm can carry mitochondrial components and even entire mitochondria (Manickam, 2022). The transport of mitochondria via extracellular vesicles or other transport mechanisms including nanotubes (Scheiblich et al., 2021), exophers (Melentijevic et al., 2017) and gap junctions (Norris, 2021) has been termed mitochondrial transfer (to be distinguished from the terms “mitochondrial transformation” or “mitochondrial transplantation “that are used for techniques to introduce exogenous mitochondria into a cellular or tissue system (Caicedo et al., 2017)) and is emerging as an important means of mitochondrial communication. The exchange of mitochondria between many different cell types has been demonstrated, for example from macrophages to neurons (van der Vlist et al., 2022), from immune to cancer cells (Saha et al., 2022), from adipocytes to cardiomyocytes (Crewe et al., 2021), and many others. The role of such mitochondrial transfer in communication across cells, however, is not yet sufficiently established.

Various still poorly understood other communication channels through electrical and calcium signals, pH flashes or coordinated oscillations of mitochondrial membrane potentials have been reported to be implicated in inter‐mitochondrial crosstalk as well (Kurz et al., 2010; Santo‐Domingo et al., 2013). Established signaling molecules for mitochondrial communication are ROS, adenosine 5′‐monophosphate (AMP), nicotinamide adenine dinucleotide (NAD^+^) and a big number of mitochondrial metabolites (including various tricarboxylic acid cycle intermediates, such as succinate and fumarate; Mottis et al., 2019).

Besides physical and metabolic cues in the cellular environment (e.g., via mitochondrial ion carriers and various transporter molecules), and direct or indirect inputs (via nuclear gene expression regulation) from cytosolic signal transduction, mitochondrial receptors for systemic signal molecules also enable mitochondrial sensing of systemic signals. Among them are steroid receptors, such as glucocorticoid, estrogen and androgen receptors, that can translocate into mitochondria, as well as membrane‐bound G‐protein coupled receptors, including angiotensin, melatonin and purine receptors (for recent review, see Picard & Shirihai (2022)). However, probably many other mitochondrial receptors exist and one recently identified example are mitochondrial cannabinoid receptors (mtCB_1_) that have been shown to be involved in the regulation of cognitive functions, such as memory (Hebert‐Chatelain et al., 2016).

Mitochondria are also involved in the generation of circulating signaling factors. For example, they are essential for the biosynthesis of circulating steroid hormones and thus play a major role in steroid hormone signaling (Miller, 2013). Moreover, mitochondrial stress leads to the release of various molecules (of both mitochondrial and non‐mitochondrial origin) into the circulation, which subsequently exert physiological actions on remote target tissues. These factors have been termed mitokines (Durieux et al., 2011), the focus of this review. While some mitokines are of nuclear origin, others are derived from mitochondria but all act in response to mitochondrial stress signals and exert biological activity affecting mitochondria at distant sites in the body. The human bona fide mitokines that are discussed in more detail below, are all robustly induced by exercise.

## 4 MITOKINES

The first references to mitokines have been made in worms. In *C. elegans*, both the neuropeptide FLP‐2 and serotonin (Berendzen et al., 2016; Shao et al., 2016) were shown to be involved in the initiation of mitochondrial stress‐related inter‐organ signaling but both were not sufficient to induce mitochondrial stress responses in distant tissues, unlike the Wnt/EGL‐20 ligand that was later reported to act as a mitokine in *C. elegans* (Zhang et al., 2018).

In mammals, fibroblast growth factor 21 (FGF21) and growth differentiation factor 15 (GDF15) are the best established mitokines but several new mitokines and mitokine‐candidates have been suggested (Kang et al., 2017; Kim et al., 2017; Lv et al., 2016). It is likely that the function of many more molecules as mitokines remains to be uncovered. Mitokines exert important intra‐cellular, autocrine and paracrine functions (Rose et al., 2017); here we focus on endocrine actions related to mitochondrial stress and adaptations. The mitokines discussed below not only function as mitokines but often exert additional functions independent of signaling mitochondrial stress. Considering this distinction is important to avoid ambiguity and clarify mitokine functions versus other roles. In this regard, we first highlight the broad functions of FGF21 and GDF15, and then focus on their mitokine actions.

### 4.1 FGF21 and GDF15

The identification and cloning of FGF21 was reported in 2000 (Nishimura et al., 2000). Unlike most other members of the FGF family, FGF21 can be released in the circulation and act as a hormone (Kliewer & Mangelsdorf, 2019). It regulates numerous metabolic processes, including adipose tissue thermogenesis, hepatic lipid metabolism and glucose uptake (BonDurant et al., 2017; Fisher et al., 2012; Markan et al., 2014). Its regulation of energy homeostasis, improvement of insulin sensitivity and induction of weight loss with the related potential in the treatment of obesity and diabetes have garnered massive scientific attention in the last years (Keipert & Ost, 2021).

Although FGF21 is produced in multiple tissues, under normal physiological conditions most of the circulating FGF21 is derived from the liver (Markan et al., 2014), corresponding to the initially reported highest FGF21 expression in liver in adult mice (Nishimura et al., 2000). Nutritional stress, in particular protein restriction (Laeger et al., 2014), increases FGF21 transcription in and release from liver (BonDurant & Potthoff, 2018).

FGF21 preferentially binds a cell‐surface receptor complex, consisting of the co‐receptor β‐klotho, regulating FGF21 targeting, and a tyrosine kinase FGF receptor (especially FGFR1c), mediating intracellular signaling (Lee et al., 2018). Important target tissues for the metabolic effects of FGF21, where β‐klotho and FGFR1c are co‐expressed, are white and brown adipose tissues. However, β‐klotho and FGFR1c have been shown to also be co‐expressed in the mouse brain, in several nuclei in the hypothalamus, area postrema and hindbrain, and FGF21 signaling there has been demonstrated to regulate for example fasting responses and circadian behavior (Bookout et al., 2013; Liang et al., 2014). In the hypothalamus, FGF21‐signaling mediated induction of corticotropin‐releasing hormone levels (Liang et al., 2014) mediates hepatic fasting responses (BonDurant & Potthoff, 2018).

#### 4.1.1 Mitokine actions of FGF21

In animal models, mitochondrial stress has been shown to induce upregulation and release of FGF21 in tissues other than liver, including from skeletal muscles (Izumiya et al., 2008; Keipert et al., 2014; Pereira et al., 2017; Tezze et al., 2017). In murine muscles, FGF21 upregulation and secretion has been demonstrated to depend on phosphatidylinosistol 3‐kinase (PI3‐kinase)/Akt1 signaling (Izumiya et al., 2008) and is upregulated upon mitochondrial stress induction, such as by mitochondrial electron transport system uncoupling or inhibition (Keipert et al., 2014; Kim, Jeong, et al., 2013), mitochondrial dysfunction following downregulation of the mitochondrial fusion factor Optic Atrophy 1 (Pereira et al., 2017; Tezze et al., 2017) and mitochondrial DNA stress in mitochondrial myopathy (Forsström et al., 2019).

Kim, Jeong, et al. (2013) reported the upregulation of FGF21 via the integrated stress response (an stress‐sensing pathway that can also be activated by mitochondrial stress) in mice with specific skeletal muscle impaired autophagy. This resulted in metabolic responses in other organs, including increased browning of white adipose tissues (Kim, Jeong, et al., 2013). Dogan et al. (2014) reported the induction of a mitochondrial stress response in cardiomyocytes upon loss of mitochondrial aspartyl‐tRNA synthetase in mouse heart that led to systemic metabolic adaptations via FGF21. Exercise‐training in mice also was shown to be cardioprotective via upregulation of FGF21 specifically in the heart (Yan et al., 2017).

FGF21 may exert its effects on distant target tissues partially via regulation of mitophagy (Oost et al., 2019), mitochondrial dynamics (Li et al., 2019) and possibly mitochondrial biogenesis. Its general metabolic and thermogenic effects may indirectly regulate mitochondrial function in different brain areas, while mitochondria in specific FGF21 target regions in the brain (based on β‐klotho and FGFR1c co‐expression, see above) likely are more directly modulated by FGF‐receptor mediated intracellular signaling. FGF21‐regulation of corticotropin‐releasing factor (Liang et al., 2014) may also be associated with mitochondrial alterations, since corticotropin‐releasing factor has been demonstrated to modulate mitochondria in neurons via NF‐κB and the mitochondrial fission factor dynamin‐1‐like protein (Battaglia et al., 2020). In adipose tissue, the autocrine action of FGF21 induces upregulation of the mitochondrial biogenesis marker proliferation‐activated receptor co‐activator 1 (PGC‐1α; Fisher et al., 2012), and via activation of adenosine monophosphate‐activated protein kinase (AMPK) and sirtuin 1 (SIRT1) improves mitochondrial oxidative function (Chau et al., 2010). Importantly, in humans FGF21 upregulation in response to exercise has been observed to be mainly due to release of FGF21 from liver and not skeletal muscle (Hansen et al., 2015). Thus, while FGF21‐involvement in signaling between liver, brain and adipose tissue is well established, the relevance of muscle‐derived circulating FGF21 remains debated outside of specific animal models. Another caveat, when comparing FGF21 signaling in mice and humans, is that in humans circulating FGF21 gets degraded by the endopeptidase fibroblast activation protein, which is not the case in mice (Dunshee et al., 2016; Zhen et al., 2016).

#### 4.1.2 Mitokine actions of GDF15

Like for FGF21 (Tezze et al., 2017), GDF15 (Chung et al., 2017) circulating levels have been reported to be upregulated upon mitochondrial dysfunction in a mitochondrial stress‐dependent manner in mouse models, leading to changes in systemic metabolism, which makes GDF15 also a mitokine as *per definitionem* (Mottis et al., 2019; Tezze et al., 2019). For this reason, it is emerging as a biomarker and potential therapeutic target for mitochondrial diseases (Fujita et al., 2015; Koene et al., 2015; Yatsuga et al., 2015).

GDF15 is a highly cell stress‐responsive cytokine of the TGF‐β superfamily and regulates appetite and metabolism (Breit et al., 2021). Its cloning and characterization was reported in 1997 and was first called macrophage inhibitory cytokine‐1 (MIC‐1; Bootcov et al., 1997). GDF15 is expressed by and secreted from different tissues and its transcription is regulated in part by regulatory proteins that are also linked to cancer, such as p53 and early growth response factor 1 (Breit et al., 2021). In addition, regulators of the cellular integrated stress response and unfolded protein response potently modulate GDF15 transcription (Chung et al., 2017; Li et al., 2018). While basal circulating levels are usually low and restricted to certain tissues including liver, lung and kidney, various physiological and pathophysiological conditions (often associated with mitochondrial stress, as recently reviewed (Johann et al., 2021)) lead to variations. Intense exercise, for example, can lead to great GDF15 release from skeletal muscle but also smoking, increasing age, injury, inflammation and many diseases (notably cancers) are associated with higher GDF15 levels in serum (Tsai et al., 2018).

The preferential receptor of GDF15 is glial cell line‐derived neurotrophic factor (GDNF) receptor alpha‐like (GFRAL). In both humans and mice, GFRAL is mainly expressed in the central nervous system with foci in the area postrema and nucleus of the solitary tract in the hindbrain, both regions involved in appetite regulation (Tsai et al., 2018).

FGF21 and GDF15 likely mediate differential systemic consequences of the mitochondrial stress response, although they have overlapping functions, e.g., improving insulin sensitivity (Kang, Choi, et al., 2021). In mice with hepatic mitoribosomal defect, FGF21 was shown to specifically ameliorate glucose clearance, energy expenditure, and thermogenesis, while GDF15 was differentially beneficial for fat mass and hepatic steatosis (Kang, Choi, et al., 2021). Differential effects were also proposed exclusively within GDF15, adding more layers of complexity. For instance, pharmacological administration of systemic GDF15 in mice led to suppression of appetite and running activity, whereas physiological induction of high‐circulating GDF15 by running, which causes mitochondrial stress, did not have the same effects (Klein et al., 2021). Thus, exercise‐induced GDF15 versus pharmacological GDF15 may affect systemic metabolism differently.

Aside from FGF21 and GDF15, a number of further nucleus‐derived candidates remain to be confirmed as mitokines, such as the mitoribosomal stress‐induced FGF21‐regulator angiopoietin‐like peptide 6 (Kang et al., 2017) or the adipocyte metabolism regulating adrenomedullin 2 (Lv et al., 2016). Besides the above mentioned molecules, which are all expressed in the nucleus, several mitochondria‐derived peptides are induced by mitochondrial stress and affect systemic metabolism (Kim et al., 2017), thus also qualifying as mitokines.

### 4.2 Mitochondria‐derived peptides acting as mitokines

Mitochondria‐derived peptides are microproteins (i.e., biologically active peptides composed of less than 100 amino acids (Saghatelian & Couso, 2015)) encoded in small open reading frames of mitochondrial DNA (for a recent review see Miller et al. (2022)). Eight such mitochondria‐derived peptides have been described and among them, humanin (HN) and the peptide mitochondrial open reading frame of the 12 S rRNA‐c (MOTS‐c) are well studied exercise‐inducible mitokines. The small humanin‐like peptides 1–6 are a class of recently discovered peptides, the functions of which partially overlap with those of humanin (Cobb et al., 2016; Miller et al., 2022). Besides exerting cytoprotective effects, attenuating oxidative stress and improving mitochondrial efficiency, they also improve systemic glucose metabolism (Cobb et al., 2016). Many further hitherto uncharacterized mitochondria‐derived peptides may exist (Miller et al., 2020) and some of them could qualify as mitokines as well, but their characterization is technically challenging and only recently has become feasible (Miller et al., 2022).

HN is a 24 amino acid polypeptide encoded by the mitochondrial 16s rRNA gene and is expressed in many tissues, including heart, brain, liver, colon, skeletal muscle, kidney, testes and vascular wall. It is a suppressor of apoptosis (Guo et al., 2003) and exerts some of its anti‐apoptotic effect through binding to cell surface receptors which in turn upregulates PI3K/AKT pathway. It also may improve mitochondrial respiration and biogenesis and it suppresses calcium overload that leads to inhibition of the Jun N‐terminal kinases and p38 mitogen‐activated protein kinases (JNK/p38 MAPK) pathways and reduced ROS production and oxidative stress (Cai et al., 2021).

The capacity to regulate cell metabolism has rendered the microprotein MOTS‐c an interesting candidate as an exercise mimetic (see below). Mitochondrial stress‐regulated MOTS‐c has been shown to inhibit the folate cycle and thereby purine biosynthesis especially in muscle, which via activation of AMPK is protective against insulin resistance (Lee et al., 2015). Upon metabolic stress (glucose restriction), MOTS‐c has been demonstrated to translocate to the nucleus, where it contributes to the stress response by regulating gene expression, including of genes associated with antioxidant defenses (Kim, Son, et al., 2018).

Based on their sensitivity to physical exercise, the health‐promoting potentials of mitokines can be exploited by related life‐style interventions, as outlined in the following section.

## 5 MITOCHONDRIAL STRESS AND MITOKINES IN EXERCISE

Exercise is associated with mitochondrial stress that—via hormetic adaptations—contributes to its health‐promoting outcomes (Hawley et al., 2014). Excessive exercise, however, can also have detrimental consequences on mitochondria (Flockhart et al., 2021). The fact that mitokine‐expression and signaling can be modulated by exercise may also imply that mild mitochondrial stress is particularly beneficial for health and healthy aging. We propose that occasional mitochondrial stress, as conferred by repeated or regular bouts of exercise, are powerful stimuli that balance mitokine activity and maintain mitokine homeostasis, thus avoiding potential resistance of target cells to mitokines (as described for FGF21 (Geng et al., 2020; Salminen et al., 2017)) and keeping levels adequately high in aging and age‐related diseases. As long as cells and tissues are resilient enough to cope with acute spikes of mitochondrial stress, exposure to more severe stress will eventually be beneficial.

Mitokines are potential mediators of beneficial exercise effects by communicating exercise‐induced mitochondrial stress intra‐ and extracellularly. Whether they are causative for these health‐outcomes, and which ones are most important, remain open questions (Woodhead & Merry, 2021). Increasing levels of some of them, for example MOTS‐c, was shown to exert exercise‐mimicking effects, including weight loss, improved oxidative stress management and insulin sensitivity, and upregulation of the mitochondrial biogenesis marker PGC‐1α (Lee et al., 2015; Yang et al., 2021). Together, these findings favor the interpretation of an important role of mitokines in mediating exercise effects.

A prominent consequence of exercise is the release of FGF21 (Kim, Kim, et al., 2013), which also occurs as a result of dietary interventions, such as dietary restriction (Hill et al., 2019) and leads to various metabolic adaptations (Tezze et al., 2019). FGF21's potential to cross the blood–brain barrier and its ability to protect blood–brain barrier function (Chen et al., 2018) implicate FGF21 specifically in brain health and exercise‐benefits on the brain (Burtscher, Millet, et al., 2021). Similarly, increased circulating GDF15 levels have been reported in exercising humans (Tchou et al., 2009) and these levels may continue to (transiently) increase even after cessation of the exercise (Kleinert et al., 2018). However, several hours after intense exercise, plasma levels decrease again and basal circulating levels of GDF15 were shown to be consistently lower in physically active people as compared to their inactive peers (Conte et al., 2020). These results were obtained in athletes after an ultramarathon (Tchou et al., 2009) or in non‐athletes after moderate to intense bicycle training (Conte et al., 2020; Kleinert et al., 2018) and similar increases of GDF15 were observed after intense rugby training (Galliera et al., 2014). It can be assumed that the kinetics of GDF15 modulation by exercise depends on intensity, exercise modality and individual characteristics of the study population. The evaluation of these parameters in future research will help to tailor training plans for optimal health outcomes.

The source of released FGF21 during exercise seems to be mainly the liver in humans with probably a higher proportion coming from skeletal muscle in mice (Tezze et al., 2019). The origin of exercise‐related circulating GDF15 remains ill‐defined but muscle also does not seem to be the main source in humans (Kleinert et al., 2018). Not surprisingly, the exercise‐induced upregulation of these mitokines contribute to exercise‐related beneficial health effects (Tezze et al., 2019), including healthy aging and longevity, possibly by maintenance of adequate mitokine levels and sensitivity to mitokine signaling.

Just like for the nuclear mitokines, circulating and muscle mitochondrial derived peptide levels, in particular of humanin (Woodhead et al., 2020) and MOTS‐c (Hyatt, 2022; Reynolds et al., 2021), have been reported to be exercise‐responsive both in mice and in humans (von Walden et al., 2021). For instance, circulating HN and MOTS‐c plus muscle MOTS‐c expression were examined in recreationally active adults after an acute bout of endurance and resistance exercise, the two major types of exercise modalities (von Walden et al., 2021). The resistance exercise bout comprised two lower body exercises at 7 repetitions maximum (RM), a moderate‐heavy resistance load (i.e., ~85% 1 RM), while the endurance exercise bout consisted of 45‐min moderate intensity cycling at 70% of the estimated aerobic exercise capacity (VO_2_max). Plasma HN significantly increased at 30‐min and 3 h after acute endurance exercise but was not affected by acute resistance loading (von Walden et al., 2021). MOTS‐c in plasma and skeletal muscle was not altered by acute endurance or resistance exercise (von Walden et al., 2021). These observations suggest that the type of exercise differentially affects systemic levels of mitochondrial‐derived mitokines, with repetitive, lower tension muscle contractions characteristic of aerobic exercise possibly eliciting elevated HN systemically more efficiently.

In another study, a different exercise strategy was used to evaluate HN response (Woodhead et al., 2020). Here, plasma and muscle HN was measured after high‐intensity interval exercise. In this exercise modality, maximal effort bouts of cycling for 60 s were alternated with recovery periods of lower intensity cycling for 75 s. A single bout of high‐intensity interval exercise significantly increased HN protein levels in skeletal muscle and plasma. In a complementary experiment, mouse skeletal muscle was electrically stimulated ex vivo to mimic the contractile activity of skeletal muscle during exercise (Woodhead et al., 2020). Stimulated mouse muscle showed a 4‐fold increase in HN protein content when measured 10 min after the stimulation protocol. These observations from human and mice suggest that HN is produced locally in skeletal muscle and may contribute to elevated circulating levels of HN. Accordingly, exercise‐induced release of HN can exert systemic effects such as improved insulin sensitivity and the regulation of weight gain and visceral fat (Woodhead et al., 2020).

Another investigation conducted by members of the same research team showed that a similar interval exercise intervention increased MOTS‐c in skeletal muscle and plasma (Reynolds et al., 2021). This finding is in contrast to the lack of change in MOTS‐c by acute endurance and resistance exercise (von Walden et al., 2021), which both differ considerably from higher intensity interval exercise performed intermittently. These studies imply a dose response relationship between training load and HN or MOTS‐c response, determined by duration, intensity, and mode of exercise (Gidlund et al., 2016).

Taken together, although the mode of action of mitokines, by which they induce systemic metabolic responses to exercise, remains to be fully explained, they appear to be important players in exercise‐related benefits, and in aging and age‐related diseases.

## 6 MITOKINES IN AGING AND AGE‐RELATED DISEASES

Mitokines are emerging as key regulators of aging processes and at appropriate levels may protect from aging‐related diseases. This notion is supported by the alterations of mitokine levels during aging. Aging is associated with various mitochondrial dysfunctions (Miller et al., 2022), including attenuated mitochondrial stress response efficiency (Lima et al., 2022). A resulting impaired mitokine signaling due to these age‐related alterations may be a target to support healthy aging and prevent associated diseases. While in non‐vertebrates, such as *C. elegans*, mechanistic associations between mitochondrial stress and longevity have been clearly established (Houtkooper et al., 2013; Mouchiroud et al., 2013), potential similar causalities in human are less well known. Particularly, the precise roles of the various mitokines in aging, remain poorly understood and several previous reviews have critically summarized the debated evidence (Fisher & Maratos‐Flier, 2016; Rose et al., 2017). Rather than systematically evaluating the large body of research on contributions of the major mitokines to aging, the objective of this section is to provide selected examples, on how dysregulated mitokine levels may lead to age‐related functional decline and diseases, in order to appraise in the next section, how important a well‐calibrated balance and regulated fluctuations of mitokines are for healthy aging.

In mice, FGF21 levels have been reported to decrease with age (Fujita et al., 2016) and overexpression of FGF21 extended mouse lifespan (Zhang et al., 2012). Conversely, plasma levels of FGF21 and GDF15 were shown to increase with aging in humans (Conte et al., 2019; Youm et al., 2016)and these elevations are closely associated with aging‐related chronic diseases, including cardiovascular and metabolic disorders like heart failure, type 2 diabetes, and neurodegenerative diseases like Alzheimer's diseases (Conte et al., 2019; Youm et al., 2016).

The increased concentrations of both FGF21 and GDF15 in humans occurred not only with aging (which by itself is associated with mitochondrial dysfunction) but also in mitochondrial diseases, suggesting that mitochondrial stress due to decreasing mitochondrial integrity may affect these mitokines' levels (Conte et al., 2019; Shpilka & Haynes, 2018; Tezze et al., 2019). Thus, it is possible that these changes merely are biomarkers for increased mitochondrial stress. However, altered mitokine levels likely also bring about changes in metabolic homeostasis, which could be causal for aging‐related functional decline. For example, FGF21 gain of function has been reported to induce bone loss in mice (Wei et al., 2012) and thus has been speculated to be involved in causing aging‐related skeletal fragility. However, other studies did not observe these effects (Li et al., 2017) and whether FGF21 negatively affects bone mass in humans is not known.

Mitokine signaling is closely associated with the regulation of energy balance (Klaus et al., 2021). For example, FGF21 plays a major role during starvation or the “energy‐deficit” period after exercise (Kim, Kim, et al., 2013) and GDF15 regulates systemic energy metabolism via anorectic actions, e.g., after high‐fat feeding (Johann et al., 2021; Patel et al., 2019). GDF15 has been shown to be upregulated by peroxisome proliferator‐activated receptor β/δ (PPARβ/δ), which led to beneficial metabolic effects via activation of AMPK and subsequently reduced endoplasmic reticulum stress and inflammation, improved glucose intolerance and fatty acid oxidation in high‐fat‐fed mice (Aguilar‐Recarte et al., 2021).

Further, circulating levels of the mitochondria‐derived peptides HN and MOTS‐c seem to change during aging but related reports are inconsistent. A negative correlation of HN and age was found in mice, monkeys and humans (Cobb et al., 2016; Muzumdar et al., 2009), but not in the senescence‐resistant naked mole rat (Yen et al., 2020), suggesting a link between senescence and HN levels. Upregulation of HN‐levels further increased longevity in *C. elegans* and protected them from toxic insults (Yen et al., 2020). In contrast, another study found increased circulating HN levels in older humans (Conte et al., 2019). In line with these results, exceptional human longevity has been suggested to be correlated with high HN‐levels (Yen et al., 2020) and also with specific polymorphisms (m.1382A>C, a common variant in Northeast Asian populations) in MOTS‐c (Fuku et al., 2015).

Metabolic actions of HN comprise improved blood glucose and insulin sensitivity, as well as enhanced glucose transporter type 4 expression in rodent models of diabetes, highlighting its potential as therapeutic targets in metabolic diseases (Lee et al., 2015; Wu et al., 2021). HN treatment of healthy, middle‐aged mice accordingly improved body composition and endocrine signaling (Yen et al., 2020). Similar to HN, the plasma levels of MOTS‐c have been reported to be reduced in obesity and diabetes (Lee et al., 2015; Ramanjaneya et al., 2019) and correcting them prevented aging‐related and diet‐induced obesity and insulin resistance in mice (Lee et al., 2015).

Growing evidence indicates a role of mitokines in modulating the metabolic‐related risk factors and disease severity for example in heart failure, which is intrinsically linked to aging (Duan et al., 2019). They have also emerged as major regulators of inflammation, another factor associated with aging processes. Chronic low‐grade inflammation indeed characterizes aging and therefore has been described as “inflammaging” (Franceschi & Campisi, 2014). While GDF15 on one hand may attenuate aging‐related inflammation (Conte et al., 2022; Moon et al., 2020), a mechanism probably involved in the pathogenesis of many age‐related diseases (Franceschi & Campisi, 2014), high GDF15 levels are associated with a more adverse outcome in heart failure patients, representing an even superior (adverse) biomarker in these patients than NT‐proB‐type Natriuretic Peptide (NT‐proBNP; Mendez Fernandez et al., 2020). In mice, an age‐related decline of FGF21 was reported in thymus (in contrast to the described increasing FGF21 plasma levels in humans) and overexpression of FGF21 protected against age‐related thymic lipoatrophy and associated deterioration of thymus‐related immune function (Youm et al., 2016).

Mitochondria‐derived peptides have also been linked to cardiovascular risk factors but circulating levels of HN and MOTS‐c were downregulated in people with coronary endothelial dysfunction (Qin, Delrio, et al., 2018; Widmer et al., 2013) and low‐circulating MOTS‐c or HN levels were associated with increased risk of cardiac events in type 2 diabetes (Ikonomidis et al., 2020) or angina patients (Cai et al., 2022), respectively. Accordingly, keeping HN levels high‐protected mice from age‐related myocardial fibrosis (Qin, Mehta, et al., 2018) and MOTS‐c treatment prevented vascular calcification and myocardial remodeling in rats (Wei et al., 2020).

Besides their roles in metabolic and cardiovascular age‐related diseases, mitokines are also becoming recognized as major players in the development of age‐related neurodegenerative diseases, such as Alzheimer's disease (Matsuoka, 2011). HN was in fact first reported as a suppressor of Alzheimer's disease‐associated amyloid beta toxicity (Hashimoto et al., 2001). Hippocampal infusion of HN later was shown to rescue memory deficits and impair long‐term potentiation by increasing the density of dendritic spines and levels of synaptic proteins like synaptophysin and synapsin 1 in a rat model of Alzheimer's disease (Chai et al., 2014). In humans, Alzheimer's disease is associated with decreased HN‐levels in cerebrospinal fluid (Yen et al., 2020). Other potentially neuroprotective effects of HN have been revealed in models of age‐related macular degeneration, in which HN inhibited caspase 3 and 4 and increased mitochondrial glutathione levels (Nashine et al., 2022).

The regulation of GDF15 and FGF21 levels is highly disease specific. While plasma GDF15 was not altered in patients with Alzheimer's disease in a recent study, it was increased in type 2 diabetes patients with and without comorbid complications (Conte et al., 2021). Those diabetic patients with complications had the overall greatest concentrations of GDF15 compared to all other groups. In contrast, plasma FGF21 was not significantly altered in Alzheimer's disease or type 2 diabetes compared to healthy aging controls (Conte et al., 2021). These specific profiles of circulating mitokines may relate to disease features inherent to each condition. Systemic insulin resistance and impaired fasting glucose are major pathological features of type 2 diabetes. The possibility exists that type 2 diabetic patients are able to increase systemic GDF15, perhaps via mitochondrial stress in liver or skeletal muscle, as a compensatory attempt to enhance insulin sensitivity and restore glucose control but were unable to concurrently increase FGF21, which also improves insulin sensitivity and hepatic steatosis common to type 2 diabetes (Kang, Choi, et al., 2021). In partial support, induction of endogenous GDF15 by the liver reduced phenotypes associated with hepatic steatosis in mouse models, and thus was suggested to be a compensatory mechanism to cope with metabolic deterioration (Kim, Kim, et al., 2018).

Much attention has been paid to the roles of GDF15 and FGF21 in cancers. The topic is complex and the involvement of these mitokines is different depending on the type and stage of cancer, individual vulnerabilities as well as on the medication; several anti‐cancer treatments also increase the levels of circulating mitokines, such as chemo‐ or ionizing radiation, which increase GDF15 levels, as summarized recently (Tsai et al., 2018). It is out of the scope of this review to provide an extensive overview on the regulation of mitokines in different cancers. However, we want to outline the dramatic mitokine‐changes observed in some cancers, in order to reflect, how mitokine‐levels may relate to normal or pathological aging.

RNA‐sequencing data extracted from large databases such as The Cancer Genome Atlas demonstrated elevated GDF15 levels in over one dozen different cancer types (Wang et al., 2020). GDF15 therefore has been suggested as an auxiliary biomarker for the diagnosis and prognosis of various cancers, including lung cancer (Cai et al., 2020), for which a good correlation with clinical stage has been demonstrated (Deng et al., 2021). The role of FGF21 in lung cancer is poorly defined, however, recent reports suggest potential tumor‐promoting effect of FGF21, since it can accelerate cell growth and migration in a SIRT1/PI3K/AKT‐dependent way in vitro (Yu et al., 2021). FGF21 has also been shown to be upregulated in lung cancer tissue sample, both on the RNA and protein level (Yu et al., 2021).

High‐tumor GDF15 was further linked with reduced overall survival in head and neck, kidney and liver cancers (Wang et al., 2020). In many cancers, altered energy metabolism is a disease hallmark and reprogramming of energy metabolism is an acquired biological characteristic that promotes cancer cell proliferation and growth in order to accommodate continued tumor propagation. Cancer mitochondrial impairment and mitochondrial stress responses including mitokine induction are also a pan‐cancer observation, and these events are proposed to associate with cancer progression (Wang et al., 2020). The induction of mitokines again may initially be a compensatory mechanism, turning into maladaptation. Mitokines such as GDF15 clearly exert positive consequences in some disease conditions, but negative effects in relation to malignancies due to possible pro‐oncogenic activity cannot be excluded. For instance, a recent investigation found that pharmacological induction of mitochondrial stress in thyroid cancer cells (by reducing OXPHOS) increased GDF15 expression (Kang, Kim, et al., 2021). Moreover, tumor expression of GDF15 associated with the mitochondrial stress response in patients with papillary thyroid carcinoma (Kang, Kim, et al., 2021). This patient group also showed elevated circulatory GDF15 levels, consistent with increased expression and secretion characteristics of mitokines (Kang, Kim, et al., 2021). Further, knockdown of GDF15 in thyroid cancer cells ameliorated cancer phenotypes such as viability, migration and invasiveness through a STAT3 dependent mechanism (Kang, Kim, et al., 2021), suggesting a benefit of GDF15 inhibition in thyroid cancer. Overall these findings suggest that GDF15 is induced by mitochondrial stress in thyroid cancer cells and functions as a mitokine through downstream STAT3 activity to promote cancer progression.

Similar mechanistic frameworks in which mitokines contribute to malignant transformation and progression have also been described for other cancers such as hepatocellular carcinoma (Lee et al., 2021), the most common primary liver cancer. An additional example of potential cancer‐related mitokine action on distant target tissues was demonstrated in prostate cancer and its interaction with osteocytes in bone cells (Wang et al., 2019). Here, prostate cancer cells caused osteocytes to produce GDF15, which in turn promoted prostate cancer cell proliferation, migration and invasion (Wang et al., 2019). This finding suggests that some cancers may propagate their own expansion by GDF15 induction in distant tissues. This may not be a shared mechanism across cancer types, however, as GDF15 inhibited proliferation of lung adenocarcinoma cells (Duan et al., 2019), and low levels of GDF15 predicted poor prognosis in patients with non‐small cell lung cancer (Lu et al., 2018). The role of mitokines such as GDF15 in cancer development and progression thus may be cancer‐specific and differentially amenable to exercise interventions and needs continued exploration and refinement.

While interesting for the treatment of obesity‐related pathologies, the appetite‐ and metabolism‐regulating properties of both GDF15 and FGF21 may be dangerous in cachexia. Cachexia is a wasting condition associated with cancer and other chronic diseases such as HIV, COPD, heart failure, and chronic kidney disease. Unintended weight loss, muscle wasting and appetite loss are some prominent features of cachexia. There has been increasing interest specifically in the role of GDF15 as a biomarker and treatment target in cancer cachexia and other conditions leading to cachexia (Albuquerque et al., 2022; Jones et al., 2018; Mulderrig et al., 2021; Nakajima et al., 2019; Patel et al., 2016; Suzuki et al., 2021). Serum GDF15 levels can indeed be elevated up to 100‐fold in some cancers (Welsh et al., 2003) and then may lead to life‐threatening anorexia and cachexia, as reviewed by Breit et al. (2021). While it is debated whether altered GDF15‐levels are a cause or consequence of cancers, their involvement in cancer anorexia has become increasingly recognized. Molfino et al. (2020) recently showed the association of GDF15 levels with anorexia in gastrointestinal and lung cancer patients, however, they did not find an association of GDF15 with muscle mass in these populations. In cell and mouse models of cancer cachexia, GDF15 in tumor‐derived exosomes was reported to directly contribute to muscle wasting (Zhang et al., 2022), and localized overexpression of GDF15 in mouse skeletal muscle by electroporation caused atrophy (Patel et al., 2016), offering evidence to link GDF15 to muscle mass regulation in addition to anorexia. Further, antibody mediated antagonization of GFRAL mitigated weight loss and preserved muscle mass in cancer cachexia mouse models with high‐circulating GDF‐15 (Suriben et al., 2020). These effects were independent of food intake and may relate to fat metabolism because lipid oxidation was high in GDF15‐induced cachexia, and this was reversed by the GFRAL antibody (Suriben et al., 2020). The involvement of GDF15 in regulating features of cachexia such as anorexia, body weight and muscle mass is evident and therapeutic development based on this mechanism remains ongoing.

In summary, while mitokine levels generally change with age, these alterations differ from mitokine to mitokine. While higher FGF21 and GDF15 levels are frequently observed at higher age and also in patients suffering from age related diseases, the opposite may be true for the mitochondria‐derived mitokines HN and MOTS‐c. It is thus possible that the location of mitokine production determines the direction of concentration changes in age and disease. Excessive production of nuclear mitokines may be a consequence of continuous mitochondrial stress in senescent or diseased cells. This may eventually be associated with chronic activation of distant target cells, leading either to detrimental metabolic strain or impaired mitokine‐signal transduction to target tissues due to resistance‐related signal attenuation (Geng et al., 2020; Salminen et al., 2017). Conversely, prolonged mitochondrial stress may impair the production of mitochondria‐derived mitokines, resulting in insufficient mitokine‐signaling. Although this dichotomy may seem plausible, it is probably too simplified, since mitokine levels are regulated differentially in specific diseases. This heterogeneity becomes especially obvious in different types of cancer as well as obesity and cachexia, where in particular GDF15 can be either protective or detrimental.

## 7 REGULATION OF MITOKINE LEVELS—ALL ABOUT THE BALANCE

While FGF21 and GDF15 have been suggested to promote the maintenance of health and longevity, it remains unclear, how these benefits are achieved. Some hypotheses have, however, been put forth and include that mitokine signaling counteracts age‐related adverse metabolic changes (Salminen et al., 2017) or that FGF21 and GDF15 control life‐span via control of the coupling of mitochondrial respiration, i.e., the ratio of protons transported across the inner mitochondrial membrane used for OXPHOS, modulating energy metabolism and oxidative stress (Klaus & Ost, 2020). These arguments are in favor for health‐promoting effects of high mitokine levels. But the high FGF21 and GDF15 levels observed in older people (Conte et al., 2019), and elevated GDF15 in type 2 diabetes patients (Conte et al., 2021) and in some cancers might also indicate that these mitokines promote aging or even disease and that thus keeping nuclear mitokine levels low may be best, maybe by increasing efficient mitokine signaling (via abolishing resistance and tolerance effects). On the other hand, is has been suggested that high levels of some mitokines may be beneficial for aging and facilitate exceptional longevity (Rose et al., 2017). Based on the evidence assembled in this review, we argue that probably maintenance of an efficient induction of mitochondrial stress responses and their communication and associated flexible increases and subsequent reductions of mitokine levels determine healthy aging.

The correlation of exercise‐induced mitokine regulation and health benefits of exercise on the one hand and the dysregulation of mitokine‐levels in age and disease on the other hand demonstrate that a delicate balance and well‐calibrated spikes of mitokine levels may be required for optimal health outcomes. Transient stimulation of mitokine release, such as during exercise, could be ideal for aging to maintain regulatory mechanisms to reduce mitokine signaling during rest. A study on older Japanese men confirms that endurance exercise over 5 weeks reduced serum FGF21 levels at rest and counteracts FGF21 resistance (Taniguchi et al., 2016). Longitudinal studies investigating mitokine‐levels before, during and after exercise over several years will be important to test this hypothesis in more detail. In addition, pharmacological interventions to reduce/abolish mitokine signaling may be beneficial against various diseases, such as cancer and could provide more information about their role in aging. As highlighted, these roles are well explored in non‐vertebrate systems, however, are likely different in mammals, and even among mammals (see the discussed divergences in mitokine levels during aging in rodents and humans). We concur with Klaus & Ost, who suggest that mitokines contribute to mediate adaptive mitochondrial stress responses (mitohormesis) to promote stress resistance and health benefits (Klaus & Ost, 2020). Some recent evidence indicates that mild mitokine stimulation is beneficial but more severe activation results in a loss of these benefits in a dose dependent manner (Tezze et al., 2019), analogous to the double‐edged effects of exercise on the immune system (Burtscher, Burtscher, & Millet, 2021). While mitokines (along with other exercise‐stimulated signaling molecules) mediate the beneficial effects of exercise in many cancers (Huang et al., 2022) and other diseases, chronically upregulated mitokine signaling, such as in several types of cancers (see above), or excessive mitokine signaling in heart failure lead to continuous stimulation of mitochondrial stress responses in different tissues. Long lasting alterations of mitokine levels, such as in aging, may blunt inter‐organ mitochondrial stress signaling and thus impair adequate systemic stress responses. Accordingly, the development of a resistance to mitokine signaling in conditions of chronic metabolic stress, analogous to insulin resistance, has been suggested for FGF21 (Geng et al., 2020; Salminen et al., 2017). Supporting this notion, in mouse heart mild mitochondrial impairment stimulated FGF21‐signalling, while severe mitochondrial stress did not, which resulted in attenuated cellular stress responses (Croon et al., 2022). It could also be speculated that this curtailment of FGF21 is a mechanism in place to protect from excessive stress‐responses. Similarly, mild mitoribosomal stress in mouse hypothalamic proopiomelanocortin neurons has been demonstrated to upregulate MOTS‐c, which coordinated responses to induce thermogenesis and a cell non‐autonomous mitochondrial unfolded protein response in distal adipose tissues and protected from obesity (Kang, Min, et al., 2021). In contrast and according to the mitohormetic principle, severe mitoribosomal stress in these neurons was detrimental and even caused obesity (Kang, Min, et al., 2021). In obese, older humans, it has been observed that exercise does not induce a circulating GDF15‐response in everybody (Zhang et al., 2019). But the individuals in which exercise led to higher plasma GDF15 had a better metabolic flexibility, increased fat oxidation and insulin sensitivity (Zhang et al., 2019), potentially indicating that maintenance of efficient mitokine‐signaling is required for healthy aging.

In summary, regular bouts of moderate to intensive (but not excessive) aerobic exercise appear to be suitable stimuli for mitokine and mitochondrial stress responses, leading to their long‐term improvement and thus to a better capacity of the organism to sense and cope with mitochondrial stress, induced for example by injury, infection or other diseases but also by intensive exercise (Burtscher, Romani, et al., 2022). Differential modulation and effects of individual mitokines by specific exercise protocols, however, need to be better characterized in future research. Efficient inter‐organ mitochondrial stress signaling might further be crucial for the regulation and synchronization of systemic and tissue‐specific metabolism and metabolic flexibility, both aspects, the dysregulation of which are thought to play major roles in age‐related diseases (Smith et al., 2018).

## 8 CONCLUSIONS

Despite great advances in our understanding of the role of mitokines in the last years and increasing recognition of the vast health benefits they confer, many unknowns remain. These include the elucidation of the main signaling routes for trans‐tissue communication, optimal levels for health promotion and potential risks. Furthermore, systematic assessment of long‐distance inter‐mitochondrial signaling in response to mitochondrial stress will likely reveal a number of hitherto unknown molecules and mechanisms that might qualify as true mitokines and will provide insights in the yet enigmatic involvement of mitokines in many (age‐related) pathologies.

At present, due to inconsistent reports, it is still not clear, how the levels of the various mitokines change during aging and if these changes counteract or accelerate age‐related functional declines. Potential confounding by exogenously increasing mitokine levels in animal models that could exert distinct effects (Klein et al., 2021) has to be considered. In addition, how regular exercise modulates alterations of mitokine concentrations during aging remains elusive. Also, whether all mitokines show similar resistance effects like FGF21, which of them are essential for healthy aging and at which levels, requires further investigation. This latter question is of particular interest in light of the observation of the astonishingly high mitokine levels reported in centenarians (Conte et al., 2019); are these high concentrations merely a marker of aging or are they protective and enable living long lives?

Better understanding of the complex interactions of mitokines among themselves and with other factors, as well as their regulation during aging and progression of chronic diseases remain important future research challenges. Especially the modulation of mitokine‐signaling in response to various types and intensities of exercise is a topic, which merits detailed further investigation that may lead to better rationales for specific training programs in healthy and diseased populations and therapeutic applications for conditions in which mitokine‐signaling is perturbed.

Together, these novel avenues of exploration will further refine and reshape our understanding of mitochondria from the traditionally viewed subordinate organelle with primarily intracellular responsibilities, such as for cellular energy demands and individual cell fate, to the more contemporary acceptance of mitochondria as major regulators also of overall organismal health/disease and aging, partly through sophisticated mitokine activity among the diverse mitochondrial functions.

## AUTHOR CONTRIBUTIONS

JB and AVK drafted the manuscript. All the authors revised the manuscript. All the authors have read and agreed to the final version of the manuscript.

## FUNDING INFORMATION

No funding was received for this work.

## CONFLICT OF INTEREST

The authors declare no conflicts of interest.
